# Hippocampal epileptogenesis in autoimmune encephalitis

**DOI:** 10.1002/acn3.50919

**Published:** 2019-10-15

**Authors:** Michele Romoli, Paraskevi Krashia, Arjune Sen, Diego Franciotta, Matteo Gastaldi, Annalisa Nobili, Andrea Mancini, Elena Nardi Cesarini, Pasquale Nigro, Nicola Tambasco, Nicola B. Mercuri, Lucilla Parnetti, Massimiliano Di Filippo, Marcello D’Amelio, Sarosh R. Irani, Cinzia Costa, Paolo Calabresi

**Affiliations:** ^1^ Neurology Clinic Department of Medicine University of Perugia Perugia Italy; ^2^ Neurology Unit Rimini Infermi Hospital – AUSL Romagna Rimini Italy; ^3^ Department of Experimental Neurosciences IRCCS Santa Lucia Foundation Rome Italy; ^4^ Department of Medicine University Campus‐Biomedico Rome Italy; ^5^ Oxford Epilepsy Research Group NIHR Biomedical Research Centre John Radcliffe Hospital Oxford UK; ^6^ Oxford Autoimmune Neurology Group Nuffield Department of Clinical Neurosciences University of Oxford Oxford UK; ^7^ Neuroimmunology Laboratory IRCCS Mondino Foundation Pavia Italy; ^8^ Department of Systems Medicine University of Rome “Tor Vergata” Rome Italy; ^9^ Neurology Clinic University of Rome “Tor Vergata” Rome Italy

## Abstract

**Objective:**

Autoantibody‐mediated forms of encephalitis (AE) include neurological disorders characterized by subacute memory loss, movement disorders, and, often, frequent, focal epileptic seizures. Yet, the electrophysiological effects of these autoantibodies on neuronal function have received little attention. In this study, we assessed the effects of CSF containing autoantibodies on intrinsic and extrinsic properties of hippocampal neurons, to define their epileptogenic potential.

**Methods:**

We compared the effects of CSF containing leucine‐rich glioma inactivated 1 (LGI1), contactin‐associated protein‐like 2 (CASPR2), and γ‐aminobutyric acid receptor B (GABA_B_R) antibodies on ex vivo electrophysiological parameters after stereotactic hippocampal inoculation into mice. Whole‐cell patch‐clamp and extracellular recordings from CA1 pyramidal neurons and CA3‐CA1 field recordings in ex vivo murine brain slices were used to study neuronal function.

**Results:**

By comparison to control CSF, AE CSFs increased the probability of glutamate release from CA3 neurons. In addition, LGI1‐ and CASPR2 antibodies containing CSFs induced epileptiform activity at a population level following Schaffer collateral stimulation. CASPR2 antibody containing CSF was also associated with higher spontaneous firing of CA1 pyramidal neurons. On the contrary, GABA_B_R antibody containing CSF did not elicit changes in intrinsic neuronal activity and field potentials.

**Interpretation:**

Using patient CSF, we have demonstrated that the AE‐associated antibodies against LGI1 and CASPR2 are able to increase hippocampal CA1 neuron excitability, facilitating epileptiform activity. These findings provide in vivo pathogenic insights into neuronal dysfunction in these conditions.

## Introduction

Autoantibody‐mediated encephalitis (AE) is a clinical syndrome with features including subacute memory impairment, neuropsychiatric symptoms, movement disorders, bilateral temporal lobe involvement on brain MRI, and epileptiform EEG abnormalities.^1^ Seizures are a prominent part of the syndrome, and often the most distinctive feature. While phenotypic associations with the individual autoantibodies are well‐described,^2^, ^3^, ^4^ distinct underlying molecular mechanisms and functional consequences are still uncertain.^5^, ^6^

Internalization of glutamate receptors has been implicated in hippocampal dysfunction leading to seizures and cognitive impairment in glutamate receptor‐related autoimmune encephalitis.^7^, ^8^ Frequent seizures and cognitive impairment are also characteristic of leucine‐rich glioma inactivated 1(LGI1), contactin‐associated protein‐like 2 (CASPR2), and γ‐aminobutyric acid receptor B (GABA_B_R) antibodies.^9^, ^10^, ^11^, ^12^ Only one study has systematically examined the functional effects of serum LGI1 antibodies on synaptic transmission and showed a facilitation in mossy fiber‐CA3 synaptic transmission.^11^ Another study reported increased neuronal excitability with CASPR2 antibodies, though not on central nervous system neurons.^13^

Here, we report the electrophysiological effects on hippocampal neurons of CSF from patients with AE associated with LGI1, GABA_B_R, and CASPR2 antibodies. All of the patients presented with seizures and cognitive impairment as first clinical symptoms and the patient‐derived CSF had different pro‐epileptogenic effects with regard to the intrinsic and synaptic properties of hippocampal neurons.

## Materials and Methods

CSF from patients diagnosed with definite antibody‐associated AE^1^ at the Neurology Clinic (University‐of‐Perugia) between January 2016 and December 2016 was used. Patients' neuroradiological findings and disease course are summarized in Figure S1 and Table S1. As a control, we used CSF from a cognitively normal age‐matched patient with chronic headache. For experimental studies, 3‐ to 5‐month‐old heterozygous C57BL/6J male mice were used, to avoid bias related to hormonal fluctuations. All experiments complied with the ARRIVE guidelines and the ethical guidelines of the European Council Directive (2010/63/EU), and received ethical approval (Italian Ministry of Health #887/2017PR).

### Immunohistochemistry, hippocampal neuron culture, and cell‐based assay

**Figure 1:**
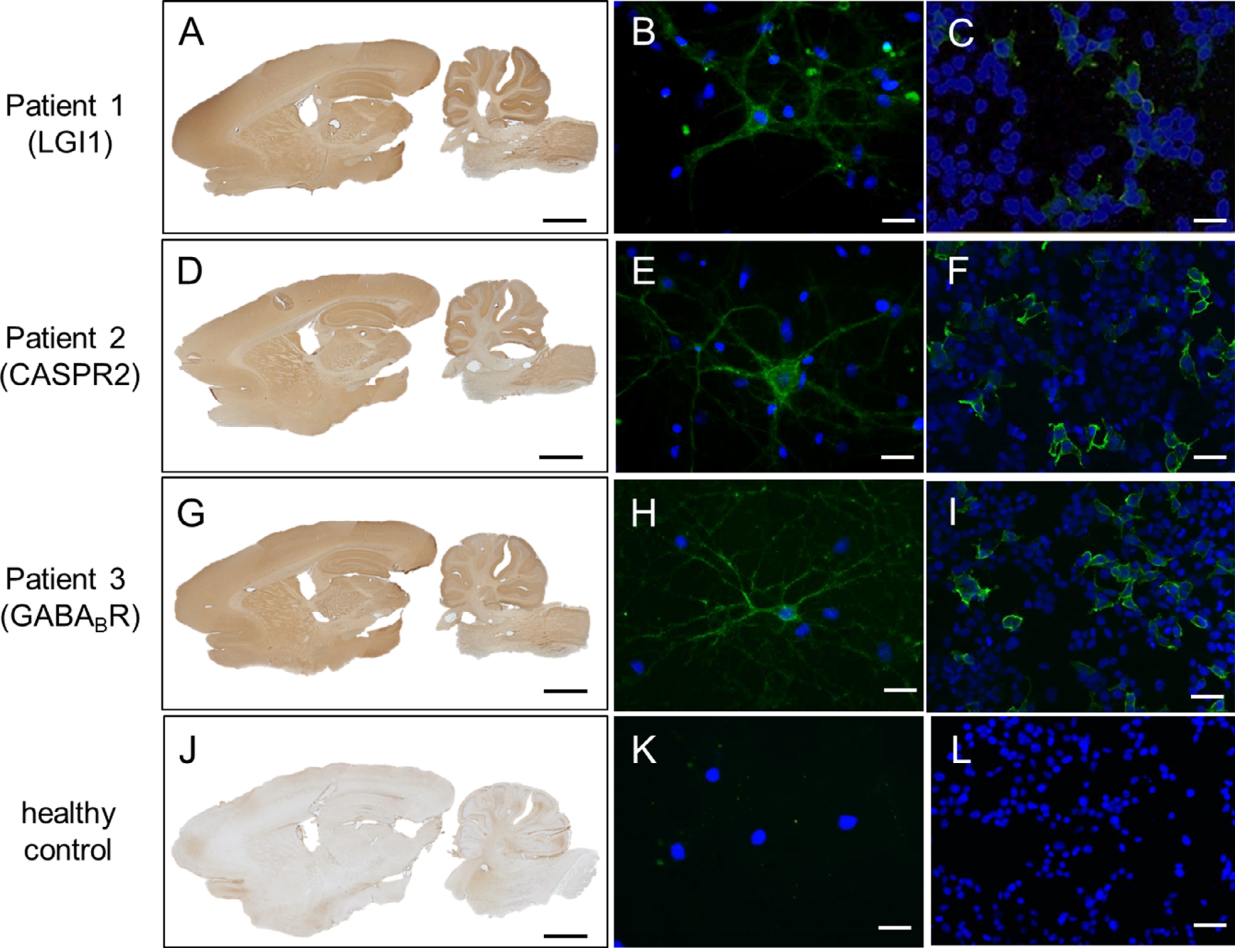
Reactivity of patient‐derived cerebrospinal fluid samples on immunohistochemistry, primary hippocampal neuron culture, and cell‐based assay. CSF antibodies to LGI1 (patient 1, A–C), CASPR2 (patient 2, D–F), and GABA_B_R (patient 3, G–I) produced neuropilar brown staining on rat brain slices (A, D, G), and green fluorescence labeling of live hippocampal neurons (B, E, H), confirming recognition of surface epitopes of the respective target protein. Protein‐specific antibody binding was confirmed on cell‐based assay for LGI1 (C), CASPR2 (F), and GABA_B_R (I) (green fluorescence). No binding was seen on rat brain slices, live neurons, or specific cell‐based assay using CSF from healthy control (J–L). Blue DAPI stains nuclei. Scale bars: rat brain slices, 2 mm; hippocampal neurons and CBAs, 20 μm. Legend – CSF: cerebrospinal fluid; pt: patient; CBA: cell‐based assay.

First, conformational antibodies were detected using immunohistochemistry on lightly fixed rat brain, optimized for neuronal surface antigens.^1^ Primary cultures of rat embryonic hippocampal neurons were established to detect whether CSF antibodies were able to recognize the native extracellular domains of surface‐expressed proteins^10^, ^11^, ^14^, ^15^. In brief, hippocampi were dissected from E17‐18 rat embryos, plated on a 12‐mm glass coverslip in a 35‐mm petri dish, and stained after approximately 21 days. Commercial‐fixed human embryonic kidney cell‐based assays (HEK‐CBA; Euroimmun‐Lübeck, Germany) expressing LGI1, CASPR2, or GABA_B_R were used, in accordance with the manufacturer's instructions, to characterize the target antigens of the antibodies. Results were visualized by a light or fluorescence microscopy (Fig. 1).

### Stereotaxic injection of human CSF in mice

Injection of human CSF into the mouse dorsal hippocampus was performed under stereotactic control. A random number was assigned for each animal, and, throughout, operators were blinded to the CSF antibody status. Mice were anesthetized with Rompun (20 mg/mL, 0.5 mL/kg; Bayer) and Zoletil (100 mg/mL, 0.5 mL/kg; Virbac;i.p.) and positioned in stereotactic apparatus before a burr hole was made under asepsis. A 2 μL mixture of human CSF with a small quantity of the tracer Fast Blue (FB; Sigma‐Aldrich) was injected unilaterally into the dorsal CA3‐CA1 regions (+1.8 AP, ±1.2 ML, −1.5 DV).^16^ The mixture was slowly infused via an internal cannula connected by Tygon tubing into a 5‐μl Hamilton needle syringe over 3 min to allow diffusion into the target brain area. Following infusion, the needle was kept in place for an additional 10 min to prevent solution backflow, before being slowly retracted. After surgery, the skin was sutured and mice were returned to their home cage and monitored during recovery. Animals were used for in vitro electrophysiological experiments 24 h following surgery. At the end of the experiments, the accuracy of the stereotactic injection was confirmed by immunofluorescence. Data from brain slices showing a misplaced injection site were discarded.

### Brain slice preparation

Acute brain slices were obtained following halothane anesthesia and decapitation. The brain was rapidly removed and parasagittal slices(300 µm) containing the injected dorsal hippocampus were obtained as previously described.^17^ Briefly, slices were cut with a vibratome (VT1200S, Leica) in chilled bubbled (95%O_2_, 5%CO_2_) sucrose‐based artificial CSF containing (in mM): KCl 3, NaH_2_PO_4_ 1.25, NaHCO_3_ 26, MgSO_4_ 10, CaCl_2_ 0.5, glucose 25, and sucrose 185 (~300 mOsm, pH 7.4). After cutting, brain slices were transferred to a holding chamber with standard bubbled artificial CSF containing (in mM): NaCl 124, KCl 3, NaH_2_PO_4_ 1.25, NaHCO_3_ 26, MgSO_4_ 1, CaCl_2_ 2, and glucose 10 (~290 mOsm, pH 7.4) and left to recover for 30 min at 32°C, followed by transference to room temperature for at least 30 min prior to usage. For recordings, a single slice was transferred to a recording chamber (volume ~ 0.6 ml), placed on the stage of an upright microscope (Axioscope 2FS; Carl Zeiss, Germany), and was continuously perfused with standard bubbled artificial CSF (2.5–3.0 mL/min). All recordings were performed at room temperature.

### Patch‐clamp recordings

Neurons were visualized using infrared differential interference contrast. Pyramidal neurons of the dorsal CA1 were identified as tightly packed cells positioned in the stratum pyramidale. For current‐clamp recordings, the electrodes (3‐6 MΩ) were pulled from thin‐wall borosilicate glass tubes and were filled with (in mM): K‐gluconate 120, KCl 20, MgCl_2_ 2, EGTA 0.2, HEPES 10, Mg‐ATP 4, and Na‐GTP 0.3 (275–285 mOsm, pH 7.4). Access resistance was monitored online throughout each experiment and recordings were discarded if either access resistance or holding current increased by more than 25% during the experiment. No liquid junction potential correction was implemented.

Current‐clamp recordings from pyramidal neurons followed predefined procedures.^17^ Briefly, short‐duration (50 ms) depolarizing current steps of increasing amplitudes (10 pA increments) were used for evaluating the action potential (AP) threshold, determined by the maximum of the second derivative of membrane potential by time, corresponding to the starting inflection point. Longer steps (600 msec, 50 pA increments) were used to obtain current‐voltage curves at subthreshold responses and AP numbers at suprathreshold responses. Membrane resistance (R_m_) was calculated from the slope after linear regression of current‐voltage curves at steady‐state subthreshold responses. Sag‐ratio was the ratio of the steady‐state versus peak potential during subthreshold responses to −200 pA current injections. Cell capacitance (C_m_) was taken online from the membrane seal test function of pClamp 9 (−5 mV step, 15 msec). Current‐clamp recordings of current‐induced spiking were obtained from resting membrane potential (RMP) kept to −70 mV by DC current injection. The RMP was evaluated by recordings with no injected current and the mean number of spontaneous APs fired/minute of recording for each neuron was calculated for 3 min of recordings, following breakthrough. Six to eight animals were used for each human CSF sample.

### Field recordings of excitatory postsynaptic potentials

Standard field responses were induced by a concentric bipolar stimulating electrode (FHC Inc.; Bowdoin, ME) placed in the Schaffer collateral pathway in the stratum radiatum and afferent stimulation (100 µsec duration) was delivered every 30s. For recording population spikes, a borosilicate glass recording electrode filled with artificial CSF was positioned in the CA1 stratum pyramidale, 200–300 μm from the stimulating electrode. Input‐output curves were obtained by measuring the amplitude of the principal population spike at increasing 10 µA steps of afferent stimulation. At least 15 sweeps were recorded at the intensity yielding a half‐maximal response and the averaged response was used for calculating the number of population spikes (with amplitude threshold set at 0.2 mV) and the total duration of the evoked response (measured from the onset of the primary population spike to the offset of the last secondary population spike). For recording field excitatory postsynaptic potentials (fEPSPs), an artificial CSF‐filled borosilicate glass recording electrode was positioned in the stratum radiatum of the CA1 hippocampal region, 200–300 μm from the stimulating electrode. Input‐output curves were obtained by measuring the fEPSP initial slope at increasing 10 µA steps of afferent stimulation. Paired‐pulse ratios (PPRs) were calculated using pairs of afferent stimuli at half‐maximal intensity at 50‐msec intervals and are expressed as peak of the second/first fEPSP. All recordings were performed with a MultiClamp 700B amplifier, using a 3–4 kHz low‐pass filter, digitized with Digidata 1322A, and computer‐saved using pClamp 9 (all from Molecular Devices, USA) at a sampling rate of four times the filter frequency. Six to eight animals were used for each human CSF sample. The target number of samples in each setting followed numbers reported in published studies.^18^

### Immunofluorescence

For FB/NeuroTrace double‐labeling, slices that were used for electrophysiological recordings were fixed in 4% paraformaldehyde in phosphate buffer (phosphate buffer [PB]; 0.1 M, pH 7.4) for 4 h, and sunk in 30% sucrose in PB at 4°C. The slices were then cut into 30‐μm thick sections using a freezing microtome, counterstained with NeuroTrace 435/455 Blue Fluorescent Nissl Stain (1:200, Invitrogen), mounted using an anti‐fading agent (Fluoromount, Sigma‐Aldrich), and examined under a confocal laser scanning microscope (LSM700, Zeiss). All images were exported in tagged image file format (TIFF), contrast and brightness were adjusted, and final plates were composed with Adobe Illustrator CS3.

### Statistical analysis

Analysis was performed using Prism (GraphPad Software‐v5). All data were analyzed with one‐way ANOVAs followed by Tukey's post‐hoc test. *P* < 0.05 indicates statistical significance (in Figures: *P < 0.05, **P < 0.01, ***P < 0.001). In figures, statistical analysis is detailed in legends, in box‐and‐whisker plots the center lines denote medians, edges are upper and lower quartiles, whiskers show min/max values, and points are individual experiments. Data are available upon request.

## Results

### CSF from AE patients does not alter the intrinsic properties of CA1 pyramidal neurons

**Figure 2:**
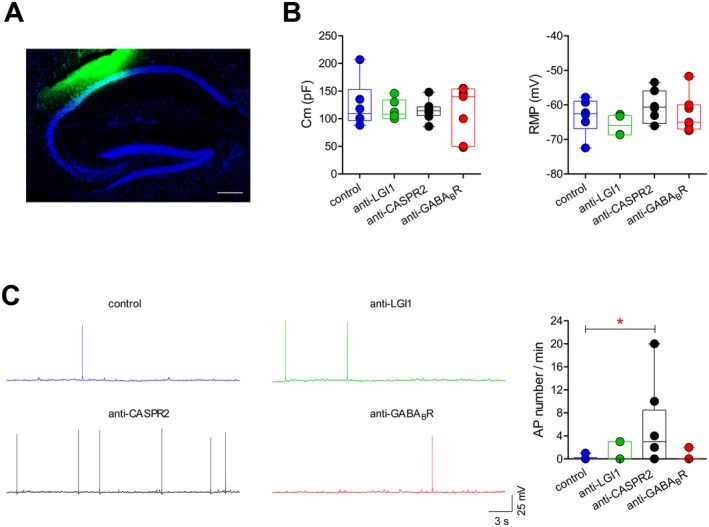
Effects of AE antibodies on membrane properties and spontaneous action potential discharge in hippocampal neurons. (A) Double immunofluorescence labelling for FB (fast‐blue; green) and Neurotrace (blue), confirming the site of injection of human CSF in the dorsal hippocampus of a male mouse. Neurotrace stains for neuronal bodies (scale bar: 200 μmol/L). (B) Membrane capacitance (Cm; left) and resting membrane potential (RMP; right) of CA1 pyramidal neurons from the dorsal hippocampus of mice injected with the indicated CSF samples (*n* = 6 neurons for control CSF; 6 for LGI1; 8 for CASPR2’ 7 for GABA_B_R. Cm: one‐way ANOVA *P* = 0.933, *F*_3,23_ = 0.144; RMP: one‐way ANOVA *P* = 0.211, *F*_3,23_ = 1.624). (C) Example traces of current‐clamp recordings showing spontaneous action potential (AP) firing in CA1 pyramidal neurons in mice injected with the indicated CSF samples. The box‐and‐whisker plot shows the number of spontaneous APs fired per min of recording (*n* = 6 neurons for control CSF, 6 for LGI1, 8 for CASPR2, 7 for GABA_B_R; one‐way ANOVA: *P* = 0.049, *F*_3,23_ = 2,564; *P < 0.05 with Tukey's post‐hoc test). In this and following figures, in box‐and‐whisker plots the center lines denote medians, edges are upper and lower quartiles, whiskers show minimum and maximum values, and points are individual experiments.

CSF samples from three AE patients and a control subject were stereotactically injected into the dorsal hippocampus of adult male mice. The co‐injection of a fluorescent tracer confirmed the accuracy of the stereotaxic injection into the CA3‐CA1 region (Fig. 2A).

We first examined the effects of the injected CSF samples at a single‐cell level using current‐clamp recordings from hippocampal pyramidal neurons in the CA1 subfield. The injection of CSF containing AE antibodies did not significantly alter the cell capacitance (Cm) or the RMP of pyramidal neurons (Fig. 2B). Only a nonsignificant trend toward more depolarized RMPs was detected with CASPR2 antibody. Though the pyramidal neurons from mice injected with the other samples only showed occasional spontaneous AP firing (control: 1/6 neurons fired single APs; LGI1: 2/6 neurons; GABA_B_R: 1/7 neurons), the majority of neurons from mice injected with the CASPR2 antibody CSF demonstrated modestly increased spontaneous AP activity (5/8 neurons; *P* = 0.049; Fig. 2C).

**Figure 3:**
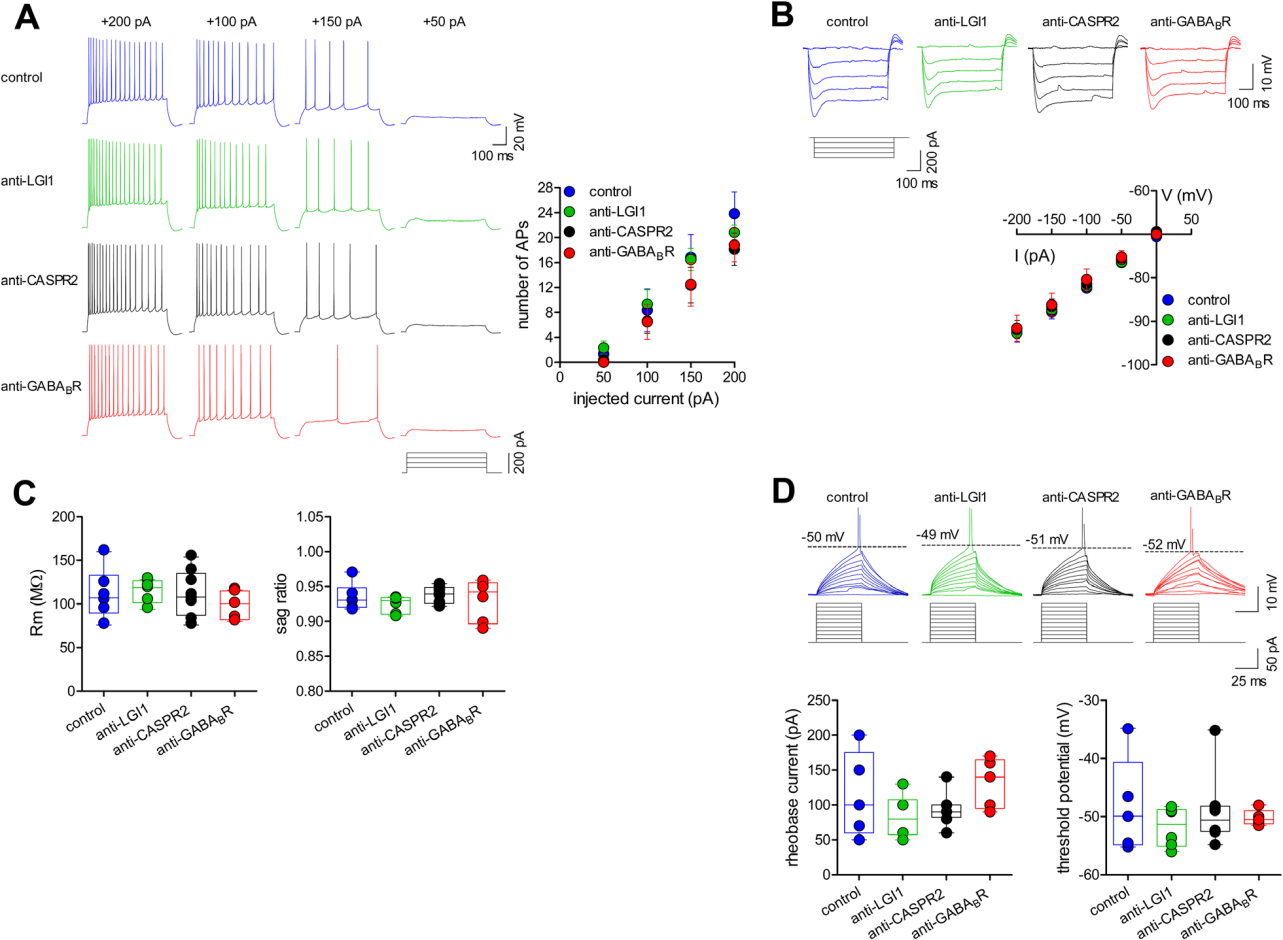
Effect of AE antibodies on evoked action potential discharge, current voltage relationship, and input resistance in hippocampal neurons. (A) Action potential (AP) firing patterns in response to 50 pA‐stepped depolarizing current injections (600 msec duration; protocol shown below) from CA1 pyramidal neurons in mice injected with the indicated CSF samples. The plot shows mean (± s.e.m) number of AP at the different input currents (*n* = 7 neurons for control CSF, 6 for LGI1, 8 for CASPR2, 6 for GABA_B_R; one‐way ANOVA: *P* = 0.403, *F*_3,23_ = 1.029). (B) Representative subthreshold responses from CA1 pyramidal neurons showing voltage responses to 50 pA‐stepped hyperpolarizing current injections (600 msec) and mean (±s.e.m; left plot) current/voltage plots for the four groups (*n* = 6 neurons for control CSF, 6 for LGI1, 8 for CASPR2, 5 for GABA_B_R; one‐way ANOVA: *P* = 0.979, *F*_3,21_ = 0.062). (C) The input resistance (Rm; center plot), calculated from the slope of current/voltage plots following linear regression, and the sag ratio (right plot) do not differ between groups (one‐way ANOVA for Rm: *P* = 0.678, *F*_3,21_ = 0.512; for sag ratio: *P* = 0.614, *F*_3,21_ = 0.613). (D) Example current‐clamp recordings (10 pA‐stepped depolarizing current injections; 50 msec), scaled to show the AP threshold, in CA1 pyramidal neurons of mice injected with the indicated CSF samples. The current needed to fire an AP is indicated below each trace. The plots show similar rheobase current (left) and AP threshold (right) for the four groups (*n* = 5 neurons for control CSF, 6 for LGI1, 8 for CASPR2, 5 for GABA_B_R; one‐way ANOVA for rheobase current: *P* = 0.175, *F*_3,20_ = 1.826; for AP threshold: *P* = 0.713, *F*_3,20_ = 0.460).

We then tested the intrinsic membrane properties of pyramidal neurons under current‐clamp conditions, following the application of depolarizing and hyperpolarizing current pulses. Long depolarizing currents elicited similar numbers of AP in all four animal groups (Fig. 3A). Analyses of responses to hyperpolarizing current pulses showed that the current‐voltage relationship (Fig. 3B), input resistance, and sag (Fig. 3C) were not significantly different between groups. Similarly, the AP firing threshold did not vary significantly across groups and similar drive current was needed to fire an AP (Fig. 3D).

### LGI‐1 and CASPR2 antibodies enhance hippocampal field potentials

**Figure 4:**
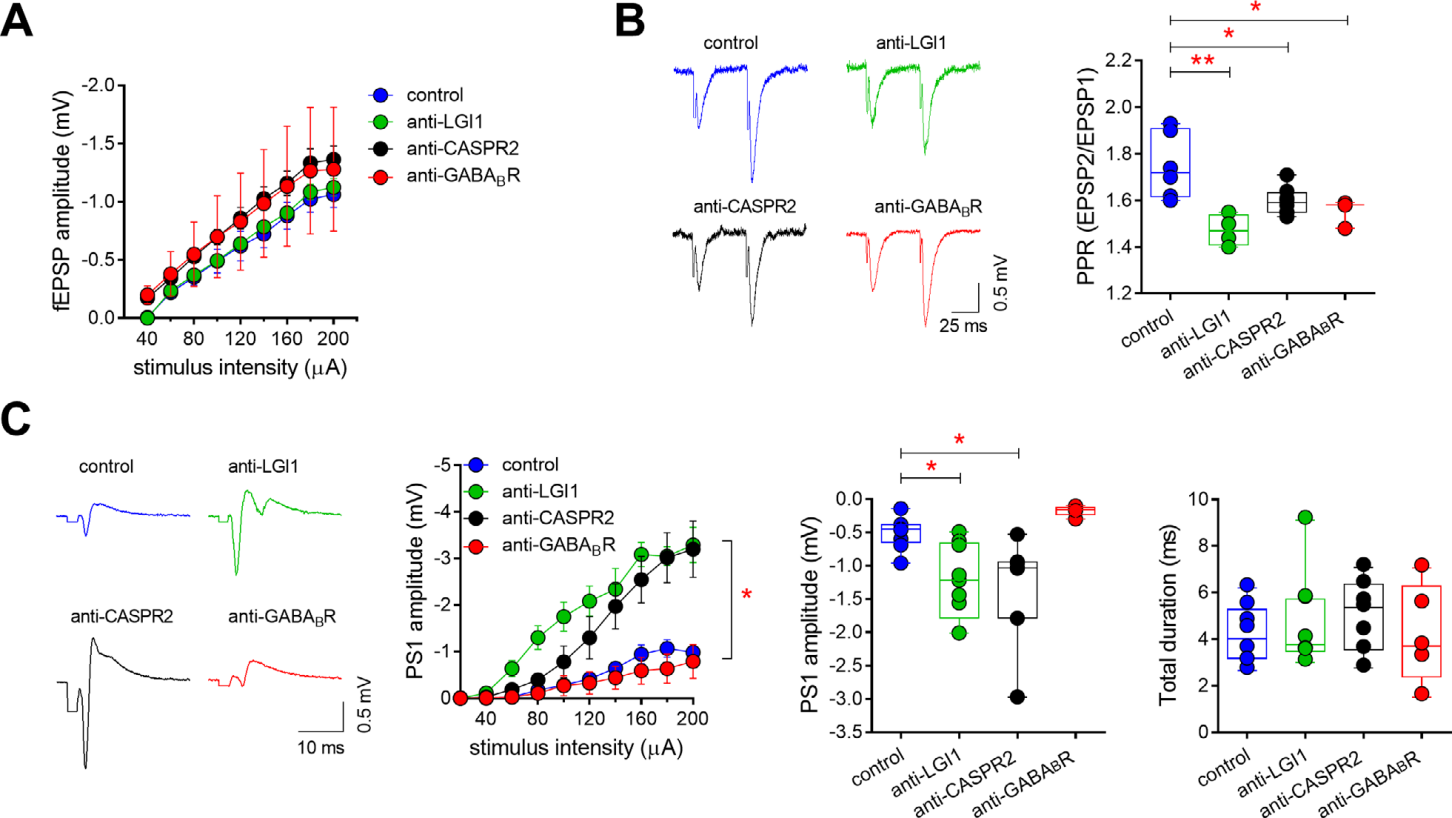
Effects of AE antibodies on input/output curves, paired‐pulse responses, and field potential features in the CA1 area. (A) Mean (±s.e.m) input/output plot of fEPSPs recorded in the CA1 stratum radiatum in response to a range of Schaffer collateral stimulation intensities (n = 6 slices for control CSF, 6 for LGI1, 8 for CASPR2, 5 for GABA_B_R). (B) Paired‐pulse responses of evoked fEPSPs (50 msec interval) recorded in the CA1 stratum radiatum following half‐maximal Schaffer collateral stimulation for each group (*n* = 6 experiments for control CSF, 6 for LGI1, 8 for CASPR2, 5 for GABA_B_R; one‐way ANOVA: *P* = 0.001, *F*_3,21_ = 8.128; control vs. LGI1: **P < 0.01; control vs. CASPR2: *P < 0.05; control vs. GABA_B_R: *P < 0.05, with Tukey's post‐hoc test). Stimulus artifacts are truncated for clarity. (C) Examples of population spikes recorded in the stratum pyramidale following half‐maximal Schaffer collateral stimulation in slices from mice injected with patient and control CSF. Stimulus artifacts are truncated for clarity. The left plot shows that the input‐output curves of population spikes are significantly increased for the LGI1‐ and CASPR2 containing CSF samples (*n* = 9 slices for control CSF, 9 for LGI1, 7 for CASPR2, 5 for GABA_B_R; *P < 0.05 for 60–200 µA intensities, compared to control CSF). Similarly, at half‐maximal intensity (center plot), the amplitude of the principal population spike is significantly increased for the LGI1 and CASPR2 samples (one‐way ANOVA: *P* = 0.0007, *F*_3,26_ = 7.815; control vs. LGI1: *P < 0.05; control vs. CASPR2: *P < 0.05 with Tukey's post‐hoc test). The total duration of the evoked responses (right plot) is unchanged between groups (one‐way ANOVA: *P* = 0.777, *F*_3,26_ = 0.368).

To further evaluate the effect of AE antibodies on hippocampal circuitry, we performed fEPSPs by directly stimulating the Schaffer collateral pathway and recording in the CA1 stratum radiatum. Although the input‐output relationship of fEPSPs was similar between groups (Fig. 4A), paired‐pulse stimulation at half‐maximal intensity revealed a reduced PPR in slices from mice injected with CSF from all the AE patients compared to control CSF (Fig. 4B). This is consistent with an increase in the probability of preexisting transmitter release,^19^, ^20^ specifically with an increased probability of glutamate release from CA3 terminals.

As increased glutamate release could result in enhanced glutamatergic transmission onto CA1 neurons, we then investigated the predisposition of these neurons to elicit multiple population spikes following stimulation of the presynaptic terminals. Orthodromic Schaffer collateral stimulation at half‐maximal intensity always elicited a single population spike in the control group and in slices from mice injected with the GABA_B_R antibody CSF (Fig. 4C). However, in slices from mice inoculated with LGI1 and CASPR2 antibodies containing CSF, secondary population spikes universally followed the principal population spike, consistent with increased epileptiform activity in these mice (Fig. 4C). Moreover, in these slices, the principal population spike was considerably larger than that seen in control slices at all stimulation intensities (Fig. 4C), while the mean duration of the total response was unaltered.

Overall, our data indicate that, in contrast to GABA_B_R, the presence of LGI1 and CASPR2 antibodies in the hippocampus enhances the probability of CA3‐CA1 glutamatergic synaptic release and that this can cause an increase in epileptiform activity of CA1 pyramidal neurons.

## Discussion

Autoantibodies targeting surface antigens are emerging as a cause of severe, but potentially treatable, diseases, including various forms of autoimmune epilepsy.^1^, ^2^ Functional blocking or destabilization of the target receptor, cross‐linking, and internalization have all been proposed to explain autoantibody effects.^12^ However, reports of antibody‐induced changes in neuronal and synaptic properties are lacking, and hence the electrophysiological pathogenesis of the clinical features remains understudied.^7^, ^11^

In this study, the first to use in vivo inoculation of CSF antibodies for neurophysiological ex vivo studies, we demonstrate that antibodies targeting LGI1 and CASPR2 are able to boost glutamatergic transmission and increase epileptic activity of CA1 pyramidal neurons. Such modifications, happening within 24 h from injection, have a clear “pro‐epileptogenic” effect, and likely represent one of the pathogenic pathways leading to seizures in LGI1 and CASPR2‐associated AE.^6^ This relative rapidity compared to other studies may be attributable to the direct hippocampal injections of whole‐CSF, which may, therefore, provide a higher throughput future model to study in vivo electrophysiological effects, also on longer time‐scale.

While molecular interactions of LGI1 antibody have been described, including ADAM22/23;^12^, ^21^ the presynaptic Kv1 channels;^10^ and/or the postsynaptic involvement of AMPARs,^11^, ^21^the reports of LGI1 antibody effect on hippocampal function are very limited. This is perhaps surprising given that structural and functional imaging studies strongly implicate this area as a focus of seizures in this condition.^22^ A single study in 2011 has shown facilitation in mossy fiber‐CA3 synapses following incubation of hippocampal slices with serum LGI1‐IgG from a single patient.^23^

In our study, we have demonstrated that LGI1 antibodies are able to boost CA3‐CA1 glutamatergic synaptic transmission, increasing epileptiform activity of CA1 pyramidal neurons. This is especially intriguing given the relatively localized CA3 atrophy seen in patients with LGI1 antibodies.^22^ Our results are also complementary to those recently reported after cerebroventricular infusion of serum LGI1‐IgG in a murine model, where patch‐clamp analysis from dentate gyrus granule cells and CA1 pyramidal neurons highlighted neuronal hyperexcitability associated with increased glutamatergic transmission and higher presynaptic release probability.^11^ Our study, which utilized whole‐CSF and compared the effects of LGI1, CASPR2, and GABA_B_R antibodies, revealed that neuronal hyperexcitability differs depending on autoantibody specificity. Since control CSF did not elicit any alteration in neuronal firing or synaptic transmission, our results suggest that such properties are directly attributable to LGI1 and CASPR2 antibodies containing CSF. Also, CASPR2 antibodies subtly perturbed intrinsic neuronal properties, increasing spontaneous AP firing in CA1 neurons. Such observations will need confirmation and refinement, but add to the pathophysiological heterogeneity between LGI1 and CASPR2 antibody patients which has already been postulated from HLA differences.^6^, ^24^

A facilitation in glutamate release was also observed for GABA_B_R antibodies. However, Schaffer collateral stimulation failed to identify clear hyperexcitability in CA1 neurons exposed to GABA_B_R Ab, for which early studies suggest no antigen internalization but altered excitability on medial temporal networks.^18^, ^25^ Studies with GABA_B_R antibodies require future attention.

Despite this study shows that Ab‐containing CSF can provoke early pro‐epileptogenic changes, our approach prevents from drawing firm conclusion on long‐term epileptogenic potential and on the impact of Ab titre. Future studies might consider multiple testing at different time points to define temporal evolution of hippocampal changes, and might implement the use of CSF with different Ab titre from large biobanks, to identify dose‐effect relationship. Nonetheless, our technical approach, using whole CSF and in vivo hippocampal stereotactic injection, might represent a feasible technique to test the effect of Ab without limiting interactions with other proteins in human CSF.

In conclusion, our study provides evidence supporting a pro‐epileptogenic modification of hippocampal neuronal excitability after exposure to human CSF containing LGI1 and CASPR2 antibodies. Both antibodies exert their action by enhancing CA3‐CA1 glutamatergic synaptic transmission, with CASPR2 antibodies also producing slight modifications in CA1 pyramidal neuron intrinsic activity. GABA_B_R Ab‐containing CSF failed to produce perturbations of synaptic functioning. Further investigations are needed to confirm these findings and identify antibody‐induced alterations in receptor expression and functioning that might lead to new treatment strategies.

## Conflict of Interest

MDF participated on advisory boards for and received speaker or writing honoraria and funding for travelling from Bayer, Biogen Idec, Genzyme, Merck, Novartis, Roche and Teva. PC receives research support from Preston and Zambon to perform preclinical investigation on drugs that are not discussed in the text. MDA. was supported by the Italian Ministry of Health (Progetto Giovani Ricercatori Project Code GR‐2011‐02351457) and by a grant from the Alzheimer's Association (AARG‐18‐566270). DF was supported by the Italian Ministry of Health ‘Ricerca Corrente’ 2017‐2019 Grant (code: RC1812C) to the IRCCS Mondino Foundation. AS has received research monies/honoraria/speaker’s fees from Bial, Eisai Europe Limited, GW Pharma, Livanova, and UCB Pharma. SRI is a coapplicant and receives royalties on patent application WO/2010/046716 (U.K. patent no., PCT/GB2009/051441) entitled 'Neurological Autoimmune Disorders'. The patent has been licensed to Euroimmun AG for the development of assays for LGI1 and other VGKC‐complex antibodies. All other authors have no competing interests.

## Supporting information


**Figure S1.** Brain imaging and disease course of limbic encephalitis.

**Table S1.** Patient clinical data.
