# Supplementary material for: Hippocampal epileptogenesis in autoimmune encephalitis
Source: Ann Clin Transl Neurol. 2019 Oct 15;6(11):2261–9. doi: 10.1002/acn3.50919 (PMC6856617; doi:10.1002/acn3.50919)
Supplement: Supplementary file 1 — Figure S1. Brain imaging and disease course of limbic encephalitis. [file ACN3-6-2261-s001.docx]

**Figure 1S**. **Brain imaging and disease course of limbic encephalitis**.

Brain axial fluid attenuated inversion recovery (FLAIR) slices from a patient with LGI1 AE. Note increased hippocampal signal bilaterally (arrowhead; A) with no restriction on diffusion weighted imaging (DWI) (B) or contrast enhancement (C). Disease course of each reported case (D).


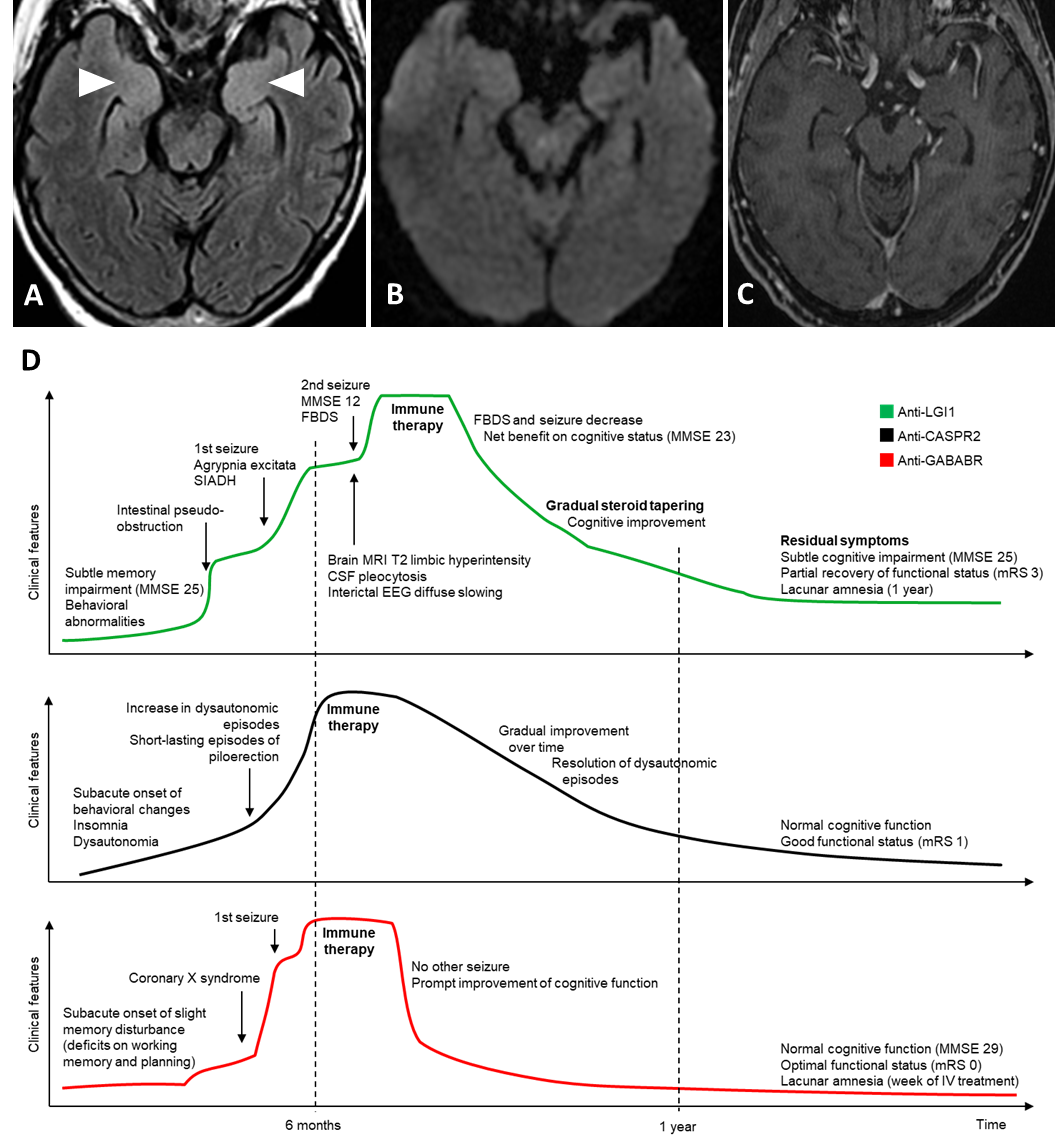


**Summary of cases**

Patient 1 was a 74 year old female who presented with two generalised seizures, faciobrachial dystonic seizures (FBDS) and a preceding history of intestinal pseudo-obstruction and cognitive decline. Brain MRI revealed bilateral medial temporal lobe hyperintensity on FLAIR sequences (Figure 1, panels A-C). Immunohistochemical analysis showed intense reactivity of the neuropil of hippocampus (Ances et al., 2005) (Figure 2A), and binding to surface epitopes expressed on cultured hippocampal neurons (Figure 2B). LGI1antibodies were identified via CBA (conventional fluorescence intensity, 3.5/5.0; CSF dilution, 1:2) (Figure 2C). After steroid plus intravenous immunoglobulin (IVIg), the patient made a clear improvement, consistent with LGI1-antibody encephalitis.

Patient 2 was a 65 year old male with a six month history of behavioural changes, subtle cognitive decline, muscle twitching/fasciculations, and sporadic episodes of piloerection. Immunohistochemistry and hippocampal neuron tests were consistent with CASPR2 antibodies (CBA end-point titrations, CSF, 1:100; serum, 1:5000) (Figure 2D,E,F). At 12 months after steroid plus IVIg course, he was independent in daily activities.

Patient 3 was a 55 year old male with a generalised tonic-clonic seizure and a 3-month history of working memory deficit and personality changes. Immunohistochemistry and hippocampal tests were consistent with GABA_B_R antibodies (CBA end-point titration, CSF, 1:100; serum, 1:800) (Figure 2G,H,I). He was treated with IVIg and steroids and returned to his normal status after 12 months (MMSE: 29).

The control subject had normal CSF findings (3 cells/mm^3, normal protein level, normal lactate).

The disease course for each patient is schematically depicted in Figure 1S (panel D).
