# Supplementary material for: Hippocampal epileptogenesis in autoimmune encephalitis
Source: Ann Clin Transl Neurol. 2019 Oct 15;6(11):2261–9. doi: 10.1002/acn3.50919 (PMC6856617; doi:10.1002/acn3.50919)
Supplement: Supplementary file 2 — Table S1. Patient clinical data. [file ACN3-6-2261-s002.pdf]

Supplementary material – Table 1. Patient clinical data.

**ID**	**Ab**	**Age**	**Onset**	**Time to diagnosis**	**Cognitive decline**	**Seizures**	**Brain MRI**	**CSF pleocytosis** (cells/ mm^3)	**Treatment**	**mRS at base-line**	**mRS at 1 year**
Patient 1	LGI-1	77	memory impairment, neuro-behavioral disturbances, agrypnia excitata, intestinal pseudo-obstruction	6 months	at onset	yes (admitted for seizure, had also FBDS)	T2-weighted bilateral mesial temporal hyper-intensity	9	IvIg + Iv steroid, followed by gradual steroid tapering	4	2
Patient 2	CASPR2	62	dys-autonomic features, fasciculations memory impairment	6 months	at onset	yes (admitted for telemetry)	Normal	15	IvIg + Iv steroid, followed by gradual steroid tapering	2	0
Patient 3	$GABA_BR$	56	isolated memory impairment	3 months	at onset	yes (admitted for first seizure)	Normal	9	IvIg + Iv steroid	2	0

Legend - Ab: antibody; CSF: cerebrospinal fluid; FBDS: facio-brachial dystonic seizures; IvIg: intravenous immunoglobulin; mRS: modified Rankin scale.
